# Phasome*It*: an ‘omics’ approach to cataloguing the potential breadth of phase variation in the genus *Campylobacter*

**DOI:** 10.1099/mgen.0.000228

**Published:** 2018-10-23

**Authors:** Jack Aidley, Joseph J. Wanford, Luke R. Green, Samuel K. Sheppard, Christopher D. Bayliss

**Affiliations:** ^1^​ Department of Genetics and Genome Biology, University of Leicester, Leicester, UK; ^2^​ Milner Centre for Evolution, Department of Biology and Biochemistry, University of Bath, Bath, UK

**Keywords:** phase variation, simple sequence repeat, *Campylobacter*, Phasome*It*

## Abstract

Hypermutable simple sequence repeats (SSRs) are drivers of phase variation (PV) whose stochastic, high-frequency, reversible switches in gene expression are a common feature of several pathogenic bacterial species, including the human pathogen *Campylobacter jejuni*. Here we examine the distribution and conservation of known and putative SSR-driven phase variable genes – the phasome – in the genus *Campylobacter*. Phasome*It*, a new program, was specifically designed for rapid identification of SSR-mediated PV. This program detects the location, type and repeat number of every SSR. Each SSR is linked to a specific gene and its putative expression state. Other outputs include conservation of SSR-driven phase-variable genes and the ‘core phasome’ – the minimal set of PV genes in a phylogenetic grouping. Analysis of 77 complete *Campylobacter* genome sequences detected a ‘core phasome’ of conserved PV genes in each species and a large number of rare PV genes with few, or no, homologues in other genome sequences. Analysis of a set of partial genome sequences, with food-chain-associated metadata, detected evidence of a weak link between phasome and source host for disease-causing isolates of sequence type (ST)-828 but not the ST-21 or ST-45 complexes. Investigation of the phasomes in the genus *Campylobacter* provided evidence of overlapping but distinctive mechanisms of PV-mediated adaptation to specific niches. This suggests that the phasome could be involved in host adaptation and spread of campylobacters. Finally, this tool is malleable and will have utility for studying the distribution and genic effects of other repetitive elements in diverse bacterial species.

## Data Summary

1. The Phasome*It* program has been deposited in Github (url – https://github.com/JackAidley/PhasomeIt/).

2. The complete dataset for the genus *Campylobacter* has been deposited in Figshare: DOI: 10.6084/m9.figshare.7066475.v1 (url –https://figshare.com/articles/Campylobacter_phasome_analysis_generated_by_PhasomeIt/7066475).

3. The complete dataset for the *Campylobacter* isolates with meta-data has been deposited in Figshare: DOI: 10.6084/m9.figshare.7066484.v1 (url – https://figshare.com/articles/Phasome_analysis_of_host_specified_Campylobacter_isolate_generated_by_PhasomeIt/7066484).

4. The genome sequence files for the *Campylobacter* isolates with metadata have been deposited in Figshare: DOI: 10.6084/m9.figshare.7066487.v1 (url – https://figshare.com/articles/Host_specified_Campylobacter_genomes/7066487).

**Impact Statement**

The genus *Campylobacter* includes multiple bacterial species found in animals and as environmental contaminants. Many of these species cause food-borne or zoonotic infections of humans. *Campylobacter jejuni* is a major agent of human gastroenteritis with the ability to disseminate from farm animals through the food chain into food products. A key feature of the adaptability of *C. jejuni* is phase variation (PV) involving ON/OFF switches in gene expression mediated by insertions and deletions of repeats in repetitive DNA sequences. We developed a computer program, Phasome*It*, in order to rapidly and reliably detect and analyse known and putative phase-variable genes in collections of genome sequences. Using this tool we demonstrate that poly-G repetitive tracts are a major source of genetic variability in the genus *Campylobacter,* with each species having a distinctive set of ‘core’ PV genes suggestive of differential selective pressures and adaptive strategies. Analysis of the contributions of PV to disease and transmission of *Campylobacter* was investigated for the genome sequences of multiple food-chain isolates but only detected a weak association between the phase-variable gene repertoire and transmission for one clonal complex. Future applications of this intuitive tool have the potential to identify whether PV is a major determinant of pathogen transmission or disease potential in a spectrum of pathogenic bacterial species.

## Introduction

Tandem DNA repeats of the same sequence, termed simple sequence repeats (SSRs) or microsatellites, are subject to high rates of changes in repeat number during DNA replication. Repeat number changes are reversible giving rise to the phenomenon of phase variation (PV) whose characteristics are high-frequency, stochastic, reversible and heritable phenotypic changes [1, 2]. Translational PV arises when an SSR is present within the reading frame with changes in repeat number inducing frameshift mutations, typically truncating the protein and producing a non-functional product. Transcriptional PV occurs when changes in repeat number of an intergenic SSR alters the activity of a promoter or other regulatory element. Both types of SSR-mediated PV are present in several bacterial species but with significant differences in the SSR types and in the phasome (defined as the numbers and types of phase-variable genes in a genome sequence) [3–6] Other types of repetitive tracts, such as the imperfect repeats present in variable number tandem repeats, have not been associated with PV due to their lower mutation rates [7].

Species within the genus *Campylobacter* have been associated with diseases, both in humans and in domesticated animals. The most commonly isolated species are *Campylobacter jejuni* and *Campylobacter coli*, which together are the leading bacterial cause of food-borne gastroenteritis in the developed world, primarily due to their ability to colonize the caeca of chickens and enter the food chain via under-cooked chicken meat [8, 9]. The impacts of other *Campylobacter* species on human disease are not as clearly defined. For example, *Campylobacter ureolyticus,* prevalent in cattle, is associated with Crohn’s disease and irritable bowel syndrome (IBS) [10] and may be responsible for a small proportion of *Campylobacter-*related gastroenteritis. Several *Campylobacter* species are commonly isolated from apparently healthy animals and the environment [11–15]. Despite this widespread distribution and clinical relevance, we have a poor understanding of how these organisms adapt to and persist in different hosts/niches.

Comparative genomics of natural *Campylobacter* populations can provide information about the genes and evolutionary processes that underlie host adaptation. For example, variation in coding DNA sequences has been related to phenotypes that potentially provide a competitive advantage in particular hosts/niches [16–18]. The genome sequence of *C. jejuni* contains large numbers of poly-G tracts, consisting of seven or more tandemly arranged G-residues, which are highly mutable and occur more often than expected by chance [19]. Tracts of eight or more repeats have been shown using immunoblotting and reporter constructs to mediate switches in gene expression for a subset of phase-variable genes in *C. jejuni* [20, 21]. Thus, poly-C/G tracts of these lengths are known to generate PV and provide *Campylobacter* with rapid access to numerous phenotypes, which may be associated with host adaptation, potential to cause disease, spread of these species and the generation of phenotypic heterogeneity following population bottlenecks [20, 21]. However, little is known about the variability of the *Campylobacter* phasomes or the differences between species and strains, and how this relates to isolate host source.

**Fig. 1.**
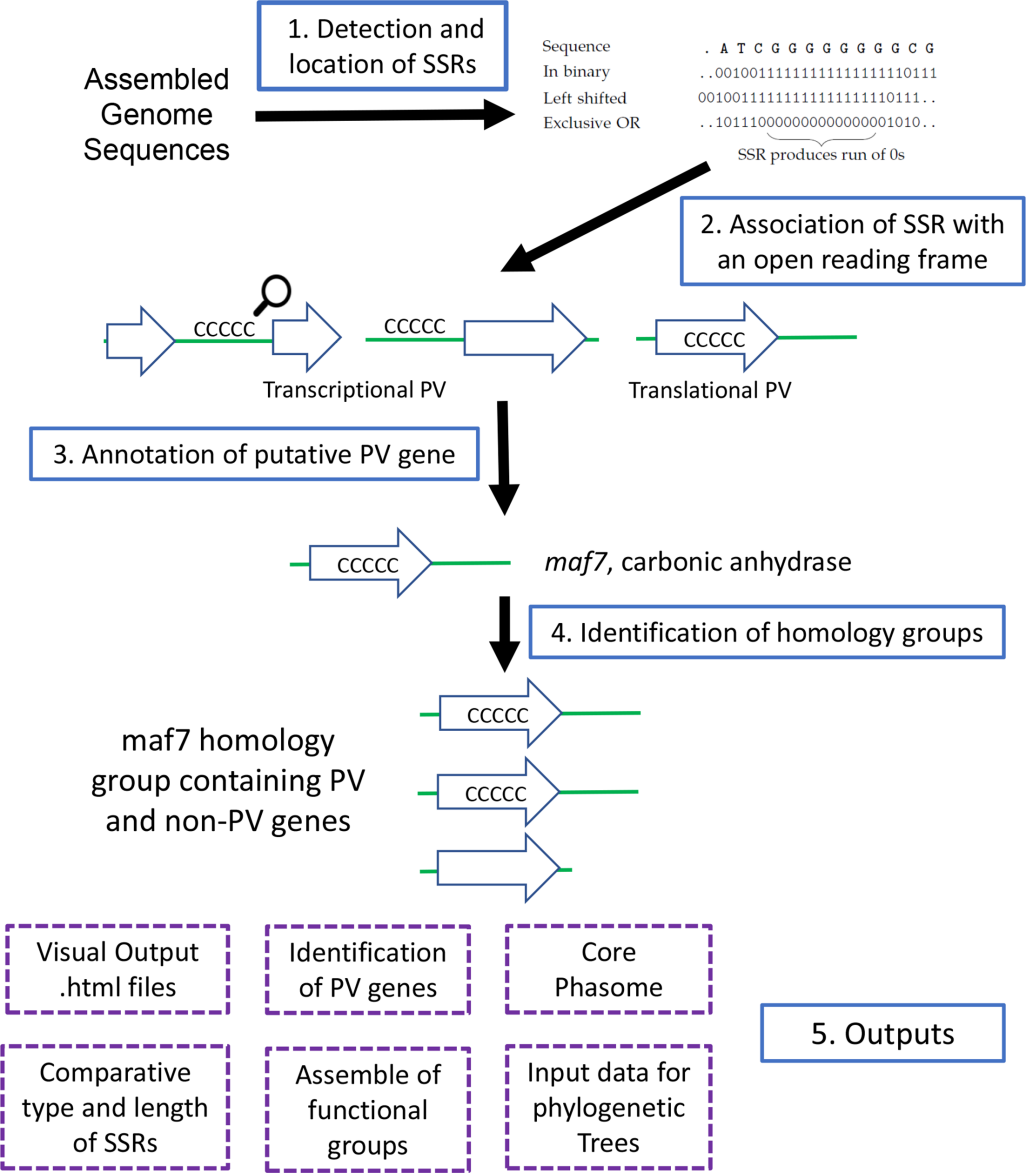
Overview of the processes and outputs of Phasome*It*. This flow diagram depicts the four major processes (steps 1–4) applied by Phasome*It* to detection of PV genes in genome sequences and the major outputs of this program (step 5). Step 1, input genome sequences are subject to a search for SSRs using Bossref that allows for user-defined inputs of repeat numbers for each repeat unit type and outputs the genome position and type of SSR. Bossref converts the DNA sequence into a binary code (2 bits per base), shifts the sequence to the left by twice the repeat unit length, performs an eXclusive OR (xor) with the original sequence, and identifies the repeats as a run of 0’s equal to twice the repeat length minus one. Step 2, Phasome*It* utilizes these SSR data to link the SSRs to genes, now defined as a phase variation (PV) gene, and to determine if the SSR mediates translational or transcriptional switching of gene expression. For transcriptional PV wherein an SSR is close to two genes, a bias (indicated by the magnifying-glass) is introduced to link the SSR to the 5′ end of a gene and hence in the putative promoter region. Step 3, Phasome*It* performs homology searches to identify known or putative functions of this gene followed by Step 4 wherein homology searches are performed against all genes in all submitted genome sequences in order to identify homology groups of related genes. Step 5, a range of output datasets (indicated in boxes) and visual outputs are generated providing both summary data and enabling interactive exploration of specific genes and specific amino acid sequences.

Here we report on a new bioinformatic program, Phasome*It* (Fig. 1), for detection and analysis of SSR-mediated bacterial phasomes from large genomic datasets. These phasomes consist of both experimentally validated phase-variable genes and genes whose PV is predicted based on having a hypermutable SSR in a position that mediates translational or transcriptional switches in gene expression. We use Phasome*It* to perform a two-stage characterization of the *Campylobacter* phasomes. First, interspecies phasome variation was investigated using 77 complete *Campylobacter* genome sequences from multiple species inhabiting diverse environmental niches. Second, 190 draft genome sequences of disease and food-chain *C. jejuni* and *C. coli* isolates were analysed for evidence of associations between phasome signatures and metadata. This catalogue of the breadth of potential PV-mediated phenotypic variation in the genus *Campylobacter* has provided new insights into evolutionary pathways underpinning the adaptability and pathogenicity of this important group of commensals and pathogens.

## Methods

### Development of Phasome*It*

Phasome*It* [Data Citation 1] can be interpreted with Python 3.5.0 (available from python.org), with both BioPython version 1.65 [22] and Natsort version 5.0.1 installed. All data generated with this program and utilized herein are available in supplementary datasets [Data Citations 2 and 3]. Genome sequences were acquired from the National Center for Biotechnology Information (NCBI) databases using Biopython. Outputs are generated in HTML 5 while JavaScript is used to dynamically alter pages. The output includes the Sorttable package, which is used under the MIT licence (http://www.kryogenix.org/code/browser/sorttable/). Bossref (for BOolean Simple Sequence REpeat Finder) was written in C++11 and compiled using GCC version 4.8.4 (for Linux) or Visual Studio 2015 community edition (for Windows).

### Identification of SRRs using Bossref

Bossref was developed for identification of SSRs from sequence data. Unlike other SSR-finding programs [23], Bossref specifically searches for repeat tracts directly relevant to PV and allows control over length cutoffs for each specific repeat type. Bossref utilizes a novel algorithm which relies on the behaviour of the simple Boolean operator ‘xor’ (eXclusive OR). The xor operator takes two binary inputs (bits) and returns 0 if they are identical and 1 if they are different. Consequently, by converting nucleotide sequences into a binary format with 2 bits for each base and comparing, using xor, with a comparable sequence left shifted by 2 bits for each base in the repeat (i.e. 2 bits for a mononucleotide repeat, 4 bits for a dinucleotide repeat, etc.), and counting the number of subsequent 0's SSRs can rapidly be pinpointed (Fig. 1). Bossref is algorithmically faster than existing programs (Table S1, available in the online version of this article), as increases in both sequence length and repeat tract length scale linearly with execution time.

### Overview of the Phasome*It* analysis process

The input for Phasome*It* is genome sequences in GenBank flat file format [24]. *Campylobacter* genomes were obtained from publicly available repositories. All the partial genome sequences used in this study are deposited in as. gbk files [Data Citation 4]. FASTA sequences are extracted and SSRs are identified using Bossref with specified cut-offs. The nearest ORF associated with each SSR is identified using the annotation data from each sequence. This is followed by a series of analyses to determine whether the SSR is within the reading frame of a gene (translational PV), within a putative promoter region (transcriptional PV) or is not involved in PV (an intergenic SSR) (Fig. 1).

Translational PV: the simplest scenario is that the SSR is present within an intact reading frame. If this not the case, as may occur for the OFF PV state, the program searches across all six reading frames for the longest ORF starting from an ATG codon that includes the repeat and accounts for possible frameshifts due to repeat tract changes.

Transcriptional PV: if the search for a translational ORF fails, the program calculates the distance from the ends of the two ORFs flanking the SSR. In order to bias this search towards promoter elements, 200 bp is added to the values for the distance between the SSR and the 3′ end of an ORF. This increases the likelihood of linking a tract with putative promoters at the 5′ end of an ORF consistent with the known locations of SSRs that generate transcriptional PV [7]. Distances are sorted and the repeat tract is associated with the closest ORF.

Note that in both cases above, Phasome*It* will associate the SSR with the nearest annotated ORF. In the absence of annotated ORFs, the program searches for novel unannotated ORFs. A conservative approach is applied when identifying novel ORFs with the requirement for a minimum ORF length of 300 amino acids that exceeds the median length of annotated proteins across several species [25]. Phasome*It* further identifies homologues of these genes in other strains by using tblastn to translate and perform homology searches with the DNA sequences (Fig. 1). The longest protein sequence generated within the three possible frames as a consequence of repeat tract length variation is used and compared using blast searches to all genome sequences in the collection. The BLOSUM-62 scoring matrix is used in combination with cut-offs of 50 % subject coverage, 40 % query coverage and an E value of 10^−6^ to both detect distant homologies and filter the results.

### Assignment of homology groups

Homology groups of PV genes are produced in a network fashion; two PV genes can be in the same group if they are both homologous to a third gene but not to each other. Non-PV genes are also identified and included in the homology groups, although they do not influence the network effect as no further blast searches are carried out on these non-PV genes (Fig. S1). Gene names or locus tags from the annotation data are used to name homology groups.

### Calling of expression states, and assignment of derivation of core phasomes

**Fig. 2.**
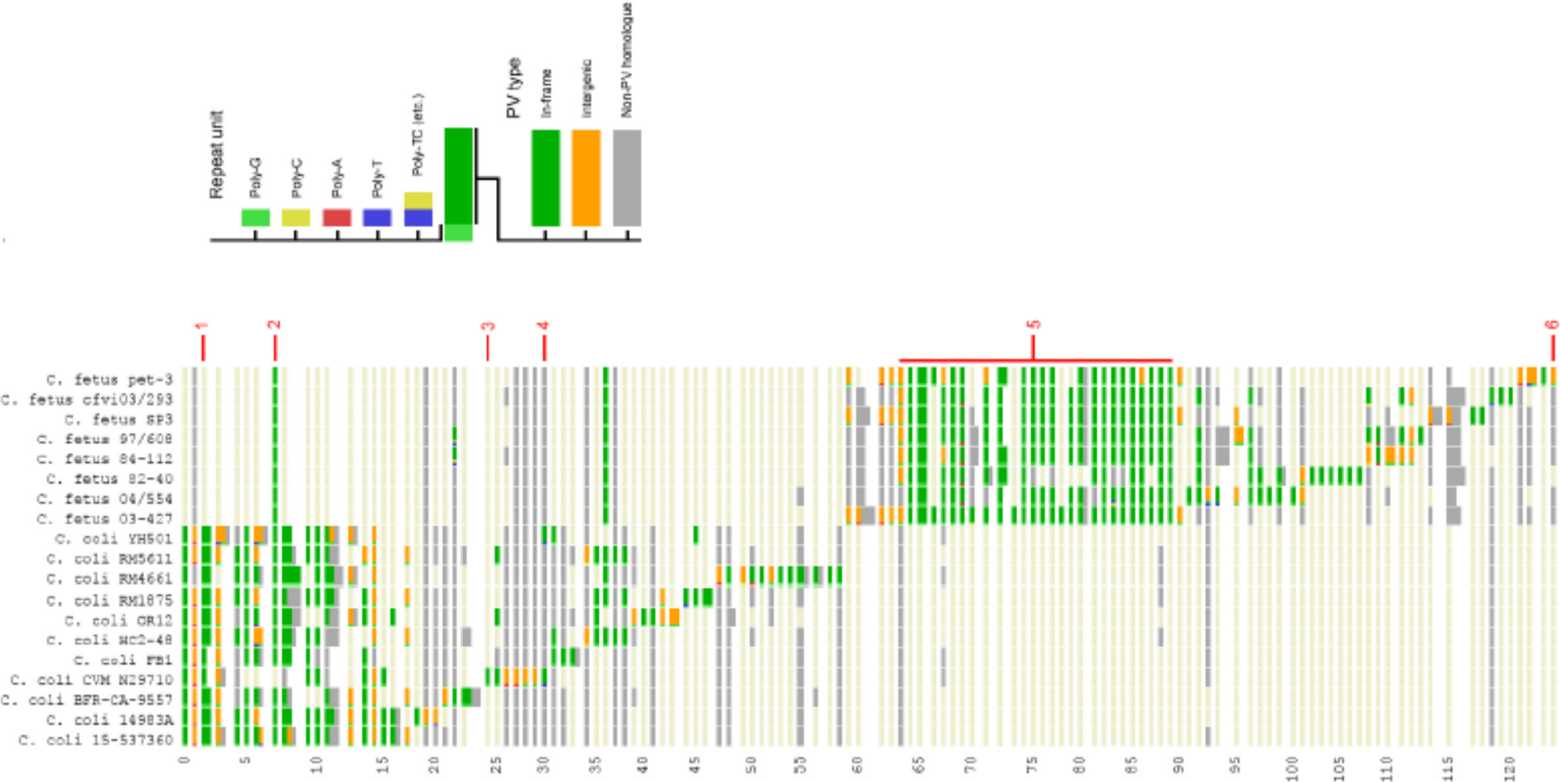
Example gene group graphic with *C. coli* and *C. fetus*. This HTML output of Phasome*It* illustrates the homology of PV genes between isolates with each numbered row representing a different homology group. Coloured blocks show the presence or absence of PV genes: green is a tract located within the ORF, orange is a tract located nearby and grey is a non-PV homologue. Coloured bars at the start indicate the repeat unit on PV tracts. Faint beige bars are present to help readability and indicate absence of any homologue in that isolate. Numbered points shown in red indicate the following: 1, PV gene conserved in all C*. coli* strains but absent in *C. fetus*; 2, PV gene conserved in all *C. coli* and *C. fetus* strains; 3, PV gene present in only one isolate; 4, PV homology group present in all *C. coli* and *C. fetus* strains but only phase-variable in one isolate; 5, block of PV genes present in the majority of *C. fetus* isolates; 6, intragenic SSR present in only one isolate.

Putative ‘ON’ lengths are calculated through comparison of the three amino acid sequences produced by potential indels within the repeat tract. The longest peptide is considered the putative ‘ON’ state for that gene while the corresponding repeat number is defined as the putative ‘ON’ length. The output produces a summary graphic and a list of all the determined homology groups and their members, which includes summary data on the rarity of each group (Fig. 2). Full visual outputs for all genome sequence datasets are available from http://jackaidley.co.uk/phasomeit. Alignment graphics from the blast matches are produced alongside trees based on the presence or absence of gene groups. Neighbour-joining trees are reconstructed using the BioPython module Bio.Phylo [26] from a distance matrix reflecting the separation between isolates, which is calculated using the Manhattan distance metric from binary lists of the presence/absence of homology groups. These data are visualized by a custom JavaScript included in the output HTML to allow dynamic re-colouring to display supplied metadata (see partial genome sequence dataset). The core phasome of a species is defined by homology groups that span large proportions of isolates and this is provided as an additional output.

### Analysis process for different genome sequence datasets

The 77 closed *Campylobacter* genome sequences were extracted from NCBI on 5 September 2016. Multiple *C. jejuni* strain NCTC 11168 sequences [27, 28], and all sequences from a large sequence type (ST)-complex 677 sequencing project except one [29], were excluded. Draft genome assemblies were kindly provided Dr Guillaume Méric for isolates sampled from several known host sources including human, cattle, chicken, wild birds and the environment (2009–2011) [16, 30], which represent potential disease source and sink populations. To this collection, NCTC 11168 [3, 31] and 15-537360 [32] were added to provide a known comparison for *C. jejuni* and *C. coli*. Extrachromosomal elements, including plasmids, were removed and analysis focused on the chromosomal genome sequence alone.

### Cut-offs applied to SSRs

The cut-off levels for repeats are configurable to the target organisms, but for the *Campylobacter* sequences analysed, the cutoffs were seven or more Gs or Cs, 10 or more As or Ts, six or more dinucleotide repeats, and five or more tetra- and penta- nucleotide repeats – consistent with tracts shown to facilitate PV in the literature [2, 21, 33–35]. Trinucleotide repeats were excluded because they cannot produce frameshift mutations.

### Statistical analysis

Statistical analyses were performed using Graphpad Prism. Statistical differences between locations of SSRs (in coding region vs. intergenic region) were determined using a z-test. Statistical significance between the numbers of PV genes in each species was determined using ANOVA.

## Results

### Development of a program for high-throughput detection and analysis of SSR-mediated phase-variable loci in genome sequence data

The basic premise underpinning development of Phasome*It* was that putatively phase-variable genes can be identified in bacteria by analysis of SSRs in genome sequence data. Another key developmental goal was a requirement for user-friendly data outputs. Phasome*It* intergrates an SSR repeat finder (Bossref) with sequence analysis and homology searches to determine whether an SSR has the potential to drive PV and to determine the putative function(s) of the associated ORF (Fig. 1). A key output is a series of HTML files, viewable on any HTML5-compatible browser, and a central index file that allows navigation of these datasets by hyperlink and contains summary information. The primary outputs are: (i) per-genome SSRs; (ii) repeat types and numbers; (iii) gene groups; and (iv) the core phasome. A file is produced for each genome sequence listing all putative PV SSRs with their key attributes, namely position, type and number of repeats, putative ON repeat numbers, and functional annotations/homologues for associated gene products.

### Poly-G/C are the most common form of putatively variable tract in *Campylobacter* species

SSR-mediated PV mediated by poly-G/C tracts is known to be highly prevalent in *C. jejuni* [21], but it was unclear if this was a specific feature of this species or occurred across the whole genus. Phasome*lt* was therefore applied to 77 *Campylobacter* genome sequences representing 14 identified species and one unidentified species (Table S2). Multiple genome sequences were included for *C. jejuni* (*n*=35), *C. coli* (*n*=10), *C. fetus* (*n*=8) and *C. lari* (*n*=7). Only one to three genome sequences were available for the other species.

Cut-offs for poly-G/C (≥7), poly-A/T (≥10), dinucleotide (≥6) and longer repeat units (≥5) were selected based on published data [2, 21, 33–35]. We identified 1944 poly-G/C tracts (91 % of all observed SSRs), 139 poly-A/T (7 %), 44 dinucleotide repeats but no longer repeat units. Most poly-G/C tracts (77 %) were located within ORFs with only 5 % having poly-C on the coding strand. Similarly, 70 % (31/44) of dinucleotide repeats were also located with an ORF. In contrast, only 50 % of poly-A/T tracts were located within an ORF, of which 81 % were poly-T on the coding strand (the difference between location of poly-G and poly-T tracts is statistically significant; *P*<0.00001, z-test). Only one species, *C. hominis*, was not dominated by poly-G tracts but instead contained two poly-G tracts and eight poly-T tracts. Dinucleotide tracts were observed in eight species but only comprised a small proportion of the total SSRs identified (between 0.1 % for *C. jejuni* and 25 % for *C. hominis*). Across all genome sequences, the poly-G/C tracts exhibited a slightly skewed distribution with 9 (53 %) and 10 (26 %) repeats forming a peak while other lengths were either less common (7, 8, 11 and 12) or rare (13–15) (Fig. S2). Poly-T tracts displayed a distinct bias towards tract lengths of 10 (i.e. the lowest detectable tract in our search), with greater lengths being infrequent (data not shown).

**Fig. 3.**
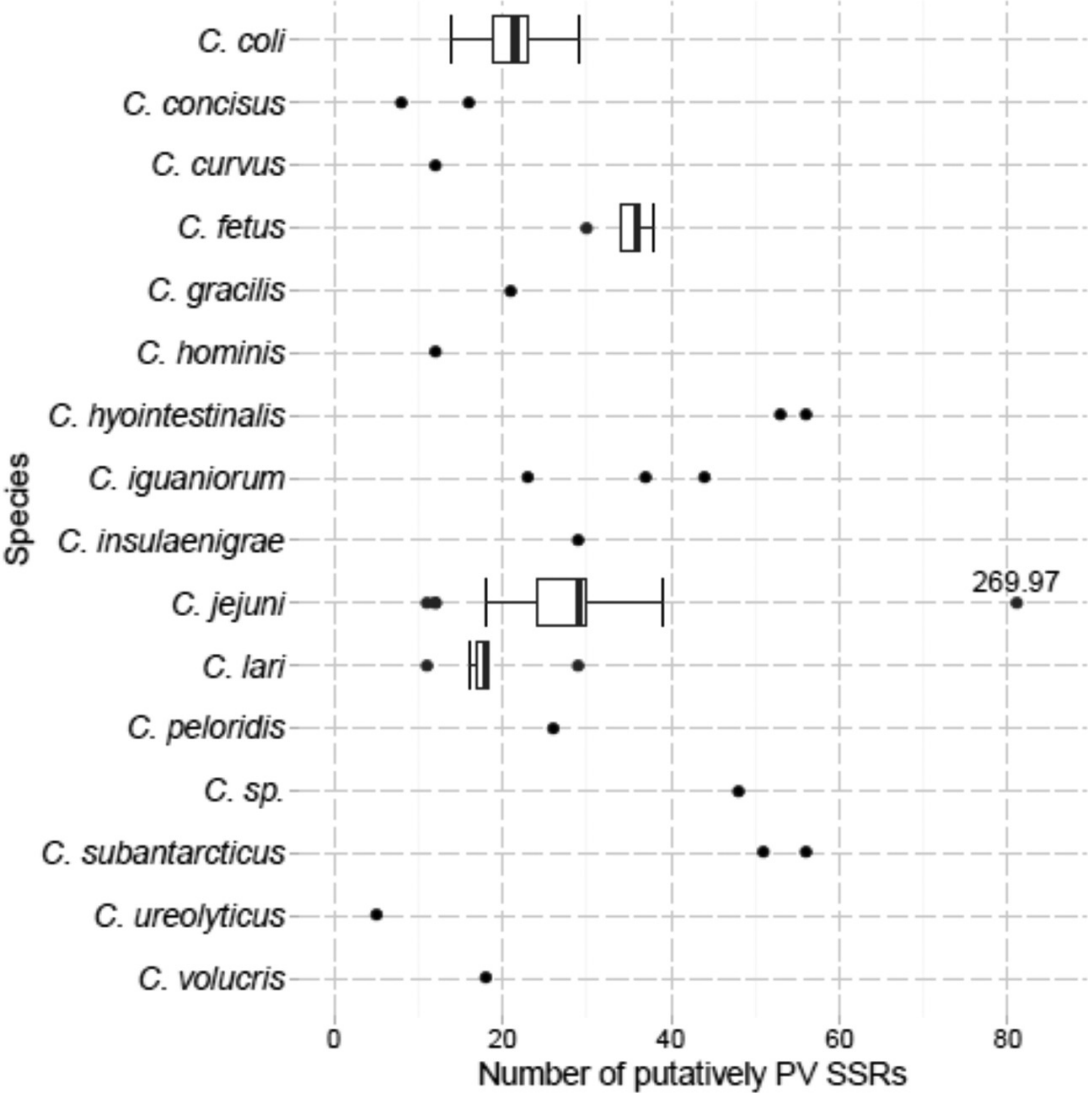
Number of PV genes per genome sequence in each species. This graph shows the number of putatively PV genes identified for each species. For species with five or more isolates, box and whiskers are shown, while for those with fewer each point is shown. These indicate the median and interquartile range (IQR) from 25 to 75 %. The whiskers stretch to the further point within 1.5 IQRs from the ends of the box, and outlying points beyond this range are individually marked. The labelled point is for *C. jejuni* subsp. *doylei* strain 269.97.

Between 5 and 81 phase-variable genes were identified for each strain with evidence of within- and between-species variation (Fig. 3). Thus, *C. jejuni* strains have a compact PV gene range of 18–39 (Fig. 3). Excluding species with fewer than three representatives, there was a statistically significant difference in the number of PV genes (*P*<0.01; ANOVA). The differences between *C. fetus* and *C. lari,* and *C. fetus* and *C. coli* were also significant (both *P*=0.018; Tukey's honest significant difference *post hoc* test). One *C. jejuni* isolate, subspecies *doylei* 269.97, has 81 putative PV-SSRs, which is 1.8-fold higher than the median for this species. Two species, *C. hypointestinalis* and *C. subantarcticus*, have higher numbers of PV genes than all other species with most of these genes containing poly-G tracts (95 and 91 %, respectively). In contrast, *C. hominis* and *C. ureolyticus* have low numbers of PV genes with only two putatively phase-variable poly-G tracts. However, only a single sequenced genome was available for each of these species.

### *Campylobacter* genome sequences feature common and multiple rare PV functional groupings

The total number of PV homology groups identified in these 77 genome sequences was 536. Two-thirds (347, 64.7 %) were found in only one isolate and a further 163 in less than 5 %. Only four PV groupings were present in >50 % of isolates. For the four species where multiple isolates were studied, a split was observed between a core number of common gene groupings and a large number of rare functional groups. This distribution is visually evident in Fig. 2 for the genome sequences of *C. coli* and *C. fetus* with a block of PV homology groups conserved across multiple isolates combined with a long tail of unique, often single-isolate PV genes.

Homology groups can consist of both PV and non-PV genes (i.e. with and without SSRs; Fig. 2). Examination of the 20 homology groups with the largest number of PV genes (i.e. ≥19) found that: 14 consisted of >80 % PV genes; 17 were found in multiple species; and three were only in one species (Table S3). Only four of these 20 gene groups (20 %), and 109 of all 536 gene groups (20 %) contained homologues with repeat tracts in intergenic regions, underlining the dominance of translational repeat-mediated PV in this species.

The three largest PV gene groupings have known or putative roles in modification of the flagellin. The *cj1295* group is involved in modification of the flagellar glycan by glycosylation with the di-O-methylglyceroyl-modified version of pseudaminic acid [36]. Similarly, homologues of the *maf1* and *maf7* groups of genes in NCTC 11168 and 81-176 produce variation of flagellar glycoforms and influence agglutination [37, 38]. Several of the other major PV groups encode transferases or enzymes with known or putative roles in modification of the lipooligosaccharide (LOS), capsule polysaccharide (CPS) or flagellum. Intriguingly two of the major groups are of unknown function with no annotation indicative of function. These groups may represent hitherto unknown functions of *Campylobacter* PV genes.

Restriction/modification (R/M) systems are found in nine PV homology groups. The most prevalent phase-variable R/M homology group was the *cj0031* group of type IIG restriction modification systems, which has homologues in every *C. jejuni*, *C. coli* and *C. lari* strain except *C. jejuni* strain 32488, but is phase variable in just 12. Another major grouping was the *cj1051c* group with phase-variable homologues in 22 genome sequences from multiple species including *C. jejuni*, *C. coli*, *C. concisus* and *C. fetus* plus 26 other non-PV homologues.

### Evidence for species-specific core phasomes

**Fig. 4.**
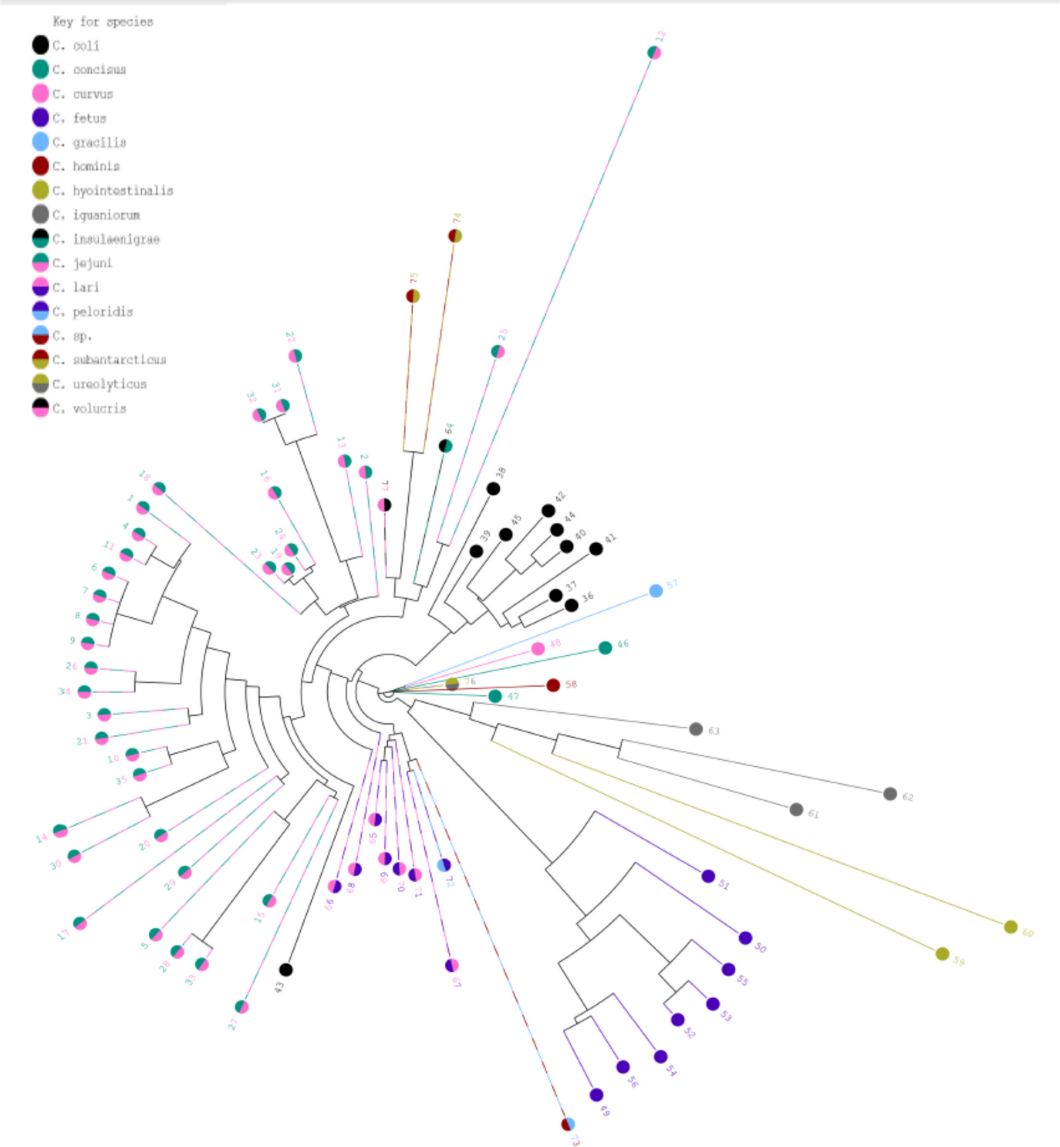
Neighbour-joining tree of phasome similarity for *Campylobacter* species. The tree depicts the phasome similarities created by applying a Manhattan distance metric to binary lists of presence and absence of homology groups. The tree is coloured by species. Numbers indicate strains, a full list is given in Table S4, and selected strains are: 1, *C. jejuni* NCTC 11168; 2, *C. jejuni* 81-176; 12, *C. jejuni* subsp. *doylei* 269.97; 25, *C. jejuni* NCTC 11351.

A phylogenetic tree based on the presence or absence of PV homology groups separated isolates by species, with clear clades for *C. jejuni*, *C. coli*, *C. lari* and *C. fetus* (Fig. 4). The *C. jejuni* clade exhibited paraphyletic clustering with one subcluster containing *C. subantarcticus*, *C. volucris*, *C. insulaenigrae* and two *C. jejuni* isolates (NCTC 11351 and 269.97 of subspecies *doylei*). This distribution indicates that PV gene groupings segregate with *Campylobacter* species, with each species having a species-specific set of conserved PV genes.

The conservation of PV genes within each species was examined by determining the core phasome, which comprises all homology groups that are present within 60 % or more of the genome sequences of a species. Table 1 shows the core phasome for *C. jejuni*, *C. coli*, *C. fetus* and *C. lari*. The core phasomes vary in size from two to 27 PV homology groups and comprise a similar proportion of all groups for *C. coli* and *C. fetus* but a much smaller fraction for *C. lari*, indicating that PV genes may be poorly conserved in this last species. Intriguingly, one of the conserved *C. lari* homology groups is also present in the *lari*-like strains of *C. peloridis*, *C. subantarcticus* and *C. volucris* with non-PV homologues in some *C. jejuni* strains. Five homology groups (*cj0045c*, *cj0170*, *cj0617*, *maf1* and *cj1295*) are shared between the core phasomes of *C. jejuni* and *C. coli*, reflecting the close phylogenetic relationship of these species.

**Table 1.** Core phasome of *C. jejuni*, *C. coli*, *C. fetus* and *C. lari*

In percentage of strains*	**Group name†**	**Function‡**	**Repeat tract§**
		***C. jejuni* (*n*=35)**	
100.0	Maf7	Carbonic anhydrase||	G (7–11)
	Cj1295	Hypothetical protein (DUF2172 domain), putative M28 family zinc peptidase||	G (7–10)
94.3	Cj0045c	Hemerythrin-like iron-binding protein	G (9–11)
88.6	CipA	Invasion protein CipA	C (8–10)
82.9	UbiE_3	SAM-dependent methyltransferase	G (8–10)
71.4	HxuB_1	Heme/hemopexin transporter protein HuxB precursor	C (8–11)
68.6	Cj1421c	Putative sugar transferase¶	G (8–11)
65.7	Maf1	Motility accessory factor||	G (7–13)
	AnsA	l-Asparaginase	C (9–11)
		***C. coli* (*n*=10)**	
100.0	Cj0045c	Hemerythrin-like iron-binding protein	G (9–11)
	Maf7	Carbonic anhydrase||	G (7–11)
90	Cj1295	Hypothetical protein (DUF2172 domain), putative M28 family zinc peptidase||	G (7–10)
	MurD	UDP-*N*-acetylmuramoylalanine–d-glutamate ligase	C (9–11)
	N149_0842	Hypothetical protein	G (9–11)
	N149_0993	Phosphoglycerol transferase	G (8–9)
	HxuA	Filamentous haemagglutinin	G (8–10)
	VacA	Autotransporter	G (8–10)
70.0	Cj0170	SAM-dependent methyltransferase	G (8–13)
	Maf1	Motility accessory factor||	G (7–13)
	lgrA	Formyl transferase domain protein	G (8–10)
	PJ17_06935	3-Oxoacyl-ACP synthase	G (7–11)
		***C. fetus* (*n*=8)**	
100	CFT03427_1684	Hypothetical protein	G (9–10)
	Cj1295	Hypothetical protein, putative M28 family zinc peptidase^5^	G (7–10)
	UbiE_3	SAM-dependent methyltransferase	G (7–10)
	CFT03427_0876	SAM-dependent methyltransferase	G (8–9)
	CFT03427_0951	Hypothetical protein	G (8–11)
	CFT03427_1021	MCP-domain signal transduction protein	G (8–10)
	CFT03427_1099	Putative membrane protein	G (9–11)
	CFT03427_1115	Autotransporter domain protein	C (8–11)
	CFT03427_1510	ATP-grasp domain protein	G (9–10)
	CFT03427_1512	SAM-dependent methyltransferase	G (8–9)
	CFT03427_1562	Probable 3-demethylubiquinone-9 3-methyltransferase	G (9–10)
	CFT03427_1573	Hypothetical protein	G (9)
	CFT03427_1574	Hypothetical membrane protein	G (9–10)
	CFT03427_1581	SAM-dependent methyltransferase	G (8–10)
87.5	MenA	1,4-Dihydroxy-2-naphthoate octaprenyltransferase	G (7–9)
	CFT03427_1442	Transformation system protein	G (8–10)
	CFT03427_1545	Radical SAM superfamily enzyme, MoaA/NifB/PqqE/SkfB family	G (9–10)
	CFT03427_1551	Short-chain dehydrogenase/reductase family protein	G (8–9)
	CFT03427_1554	Methyltransferase	G (8–9)
	CFT03427_1558	Radical SAM superfamily enzyme, MoaA/NifB/PqqE/SkfB family	G (9)
	CFT03427_1559	Hypothetical protein	G (8–9)
	CFT03427_1565	Hypothetical protein	G (9)
	CFT03427_1566	Hypothetical protein	G (9–10)
	CFT03427_1577	Hypothetical membrane protein	G (9–10)
75.0	CFT03427_1556	Formyltransferase domain-containing protein	G (9–10)
62.5	CFF04554_0871	Putative type II secretion system protein	G (9)
	CFF04554_1255	4HB_MCP sensor-containing MCP-domain signal transduction protein	G (8–9)
		***C. lari* (*n*=7)**	
100.0	UPTC4110_0710	Hypothetical protein	G (9–11)
71.4	UPTC4110_1471	MCP-domain signal transduction protein	C (9–11)

*Homology groups present in 60 % or more of the genome sequences from each species with the actual percentage for each homology group being shown in the first column.

†Each group is assigned a name based on the first gene name found in the PV genes in the group, or failing that the first locus name found. These names are preferentially chosen from a manually curated order that favours well-studied species.

‡The functional assignment is based on annotation data associated with the genome sequences and is automatically obtained as described in the main text.

§Indicates the repeat tract associated with at least 90 % of genes in each function group, and the range of repeat numbers are given in parentheses.

||Known or putative flagella-modifying protein.

¶Known or putative capsular-modifying protein.

### Variable conservation of PV within a group of homologous genes

A high proportion (56 %, 298) of PV homology groups contain both PV and non-PV homologues, indicating variable conservation of the SSR responsible for the switches in gene expression. Non-PV homologues can have high levels of sequence similarity (>90 %) but either differ at the site of the SSR (i.e. contains a shorter or interrupted poly-G tract) or have no sequence resembling an SSR (Fig. S3). Several homology groups were present in all strains but only phase-variable in one. For example, the homologue of *cj1120c* in 296.97 has an 11T tract located near the start of the gene which is absent in other isolates, although this gene is present in every *Campylobacter* genome sequence. This sporadic occurrence of PV alleles may be due to formation by mutation and weak selection against the tract, evidence of a strain-specific selective advantage, or horizontal gene transfer of variable loci into isolates lacking SSRs at these sites.

### Association of phasomes with *C. jejuni* and *C. coli* clonal complex with host source

*Campylobacter* is a zoonotic bacterium transmitted from animal reservoirs to humans and is rarely transmitted from human to human. Epidemiological investigations have shown that gastrointestinal disease due to *C. jejuni* and *C. coli* has associations with particular clonal complexes and is often associated with specific host sources [39, 40]. To investigate whether there are associations between phasome and epidemiological metadata, specifically host source, Phasome*It* was applied to a dataset consisting of a large number of non-closed genome sequences of *C. jejuni* and *C. coli* isolates of known sources.

Poly-G tracts comprised the largest proportion of identified SSRs (88 %, 3193 of 3611) with 82 % located in ORFs, similar to the multispecies genome sequence analysis. Poly-G tract lengths were also similar in distribution, with 52 % 9G and 30 % 10G (data not shown). However, the proportion of tracts in the ON state was lower than in the complete genome sequence set and closer to the proportion predicted by neutral drift [5]. The number of PV tracts per genome sequence was similar to that observed in the complete, closed genome sequences, with a median of 20 (IQR 16–26) for *C. jejuni* and a median of 15 (IQR 11–17) for *C. coli.*

**Fig. 5.**
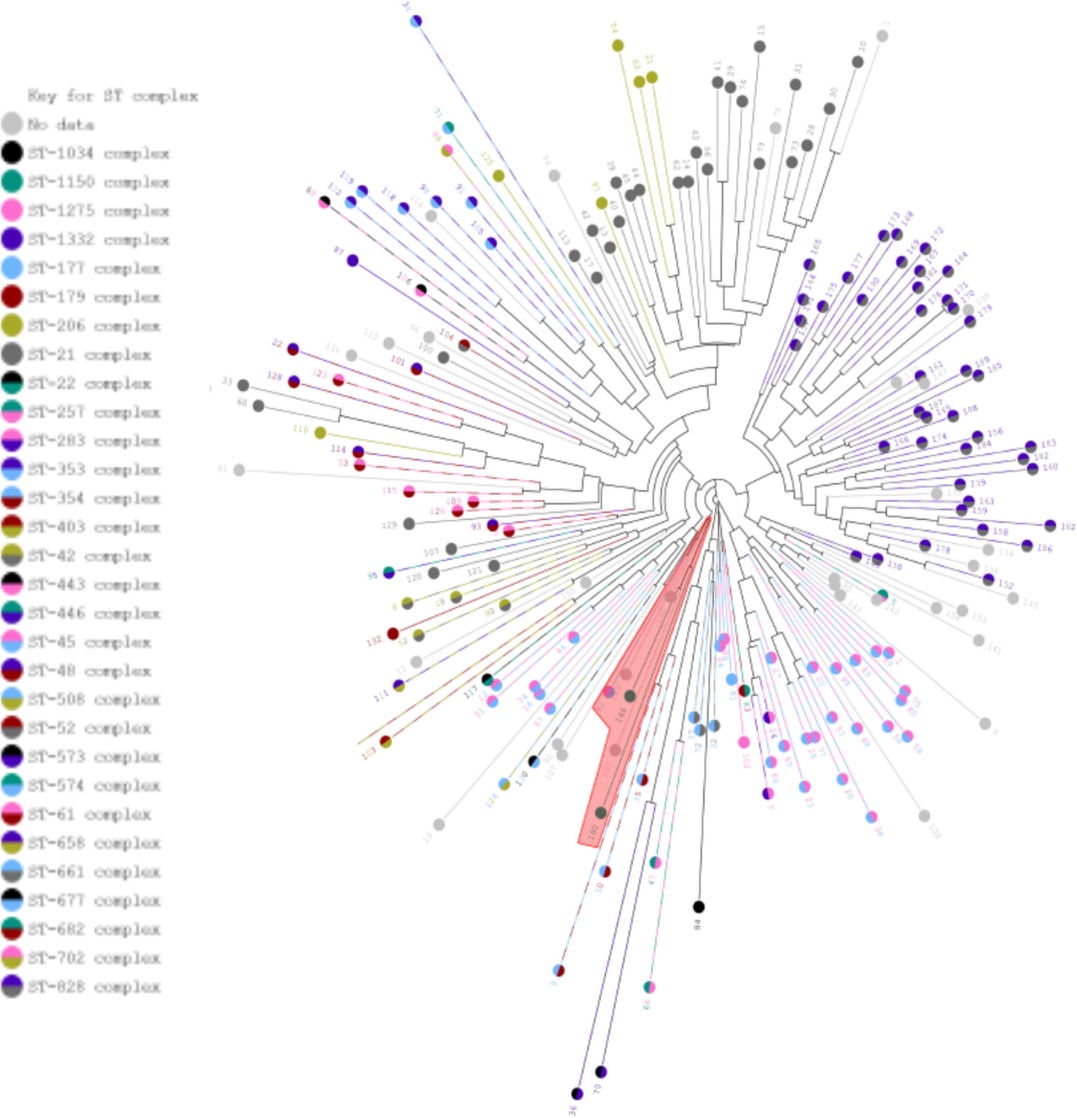
Tree based on homology groups shows clear separation by ST-complex. A neighbour-joining tree was derived using a Manhattan distance between isolates based on the presence or absence of homology groups. The tree is coloured to indicate the ST-complex for each isolate. A group of genomes with poor coverage across the highly conserved PV genes is indicated in red. Numbers are isolate numbers and carry no biological significance (see Table S5).

**Fig. 6.**
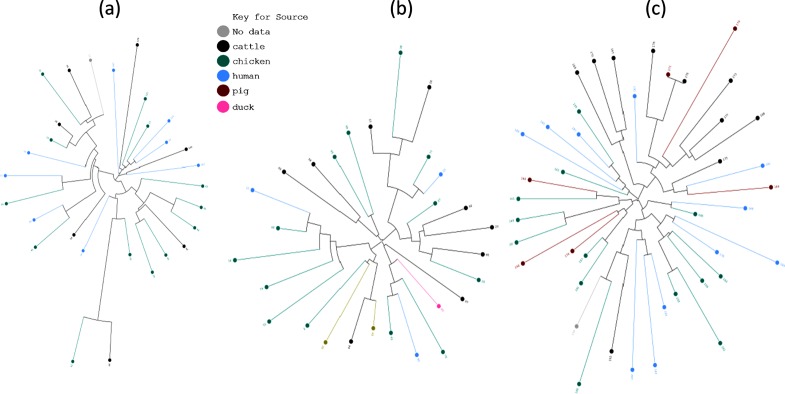
Host association in the *C. jejuni* STs. A neighbour-joining tree (derived as for Fig. 5) is shown for *C. jejuni* isolates from the ST-21 (a), ST-45 (b) and ST-823 complexes (c). Tree branches are coloured according to the source for each isolates. ST-828 isolates from cattle show significant grouping within the tree (*P<*0.05, tree-based scan statistic with conditional Poisson model). All other differences were non-significant.

Large numbers of *Campylobacter* strains have been genotyped, from various sources, by multilocus sequence typing (MLST), in which seven housekeeping loci are sequenced to obtain a seven-locus allelic profile that differentiates strains. This provides considerable information about how lineage segregation relates to strain ecology and epidemiology. For example, some clonal complexes are strongly associated with particular hosts such as chicken or cattle, while others are commonly found in both of these host species as well as in wild birds [39, 40]. Phylogenetic trees, reconstructed based on the presence/absence of PV homology groups, showed clear separation by species (data not shown) and a strong separation by clonal complex (Fig. 5). This indicates that PV genes follow a distribution related to MLST genes. One small group of genome sequences contained a mix of species and clonal complexes but was found to encompass isolates with poor assembly of conserved PV genes. Phylogenetic trees were reconstructed by the same method for three common disease-associated clonal complexes (ST-21 complex, *n*=28; ST-45 complex, *n*=28; and ST-828 complex, *n*=41; Fig. 6). These trees were then annotated with the source of each isolate (human, chicken, pig or cattle). No clusters of human isolates were detected, indicating that disease is not associated with a specific combination of PV homology groups. Clustering by host source was not observed for ST-45 or ST-21 strains. However, statistically significant clustering was detected for ST-828 isolates (*P<*0.05, tree-based scan statistic with conditional Poisson model). One cluster consisted of 14 isolates with nine from cattle, three from pigs and two from humans while the other cluster of 27 isolates contained 13 chicken, three pig, one cattle, one unknown and nive human isolates. This suggests that the phasome of ST-828 isolates may be linked with or is a genetic determinant of species specificity and the ability of chicken isolates to transmit through the food chain and cause disease in humans.

## Discussion

With the increasing availability of large bacterial genome sequence datasets there is a need for flexible bioinformatic tools that can detect and interrogate the genetic determinants of phenotypic traits. The Phasome*It* suite and display system allows cataloguing common sequence elements in genomic sequences. The principal advantages of this approach are the rapid processing times, the flexibility of search criteria, multiple output tables and a visual, searchable output window (Figs 2 and 3). The underlying algorithm can be modified to search for any specific sequence (e.g. Sigma promoter sequences) and identify the coding sequences associated with that sequence. Here, we applied Phasome*It* to investigate the number, putative function and distribution of known and putative SSR-mediated phase-variable genes across a broad range of *Campylobacter* species.

Phasome*It* detects SSRs of defined repeat number and associates these SSRs with specific genes. The cut-offs for the repeat numbers were based on experimental data from *C. jejuni* and other bacterial species that have demonstrated evidence of reversible switches in gene expression for a range of repeat numbers and repeat units for a small subset of loci (see Introduction). Genes exceeding these repeat numbers are, therefore, high likely to exhibit PV if the repeat tract is located in an appropriate position (i.e. within the reading frame or promoter). Additionally, evidence for PV has come from observations of variability in the repeat tracts between longitudinal isolates of a single strain; evidence of this type is available for several strains including all of the poly-G/C tracts with eight or more repeats of *C. jejuni* strain NCTC 11168 [41, 42]. The approach of using cut-offs enables detection of a comprehensive set of *Campylobacter* PV genes without having to validate PV for each individual gene. It should be noted that the definition of a phase-variable gene is not exact and a continuum exists from genes of low to high mutability. The cut-offs for SSRs used here included sequences where mutability may be lower than 1×10^−5^, and hence may be inefficient for the reversible ON/OFF switching hallmark of PV. This program may also misattribute the ON state of phase-variable genes if the ATG does not match the defined search criteria. Additionally, no expression states are assigned for the 25 % of *Campylobacter* SSRs located outside of any ORF as these tracts may impact the efficiency of binding of transcription factors and hence it is difficult to predict how variation in these SSRs may influence gene expression levels [1, 4, 7]. Finally, in NCTC 11168, *cj0170* has a tract located at the end of the gene, resulting in up to 22 amino acids difference in product length; however, it is not the longest product that is functional but rather the shortest [43]. These limitations are, however, minor in scope and do not undermine the major findings on PV in *Campylobacter* nor limit the application of Phasome*It* to other species but rather underline the need for experimental work to inform bioinformatic analyses.

Another potential limitation for the analysis of SSR-mediated PV is the quality and type of genome sequence data. The sequencing platform can influence and potentially compromise the quality of sequences across an SSR [6]. However, the vast majority of whole genome bacterial sequences, including all of the non-closed genome sequences utilized herein, has and is being generated on Illumina platforms. We have shown for the *Neisseria* SSR phasomes (a combination of mono-, tetra- and penta-nucleotide repeats) that there is a trend for detection of greater numbers of SSRs containing loci with increasing Illumina read length but no significant differences between the closed and partial genome sequences [6]. A similar analysis for the closed and multiple partial *Campylobacter* genome sequences of *C. jejuni* strain NCTC 11168 indicated that 96 % (32/33) of the SSR-mediated PV loci present in the closed genome sequence are detected in partial genome sequences with <400 contigs (data not shown). This high detection rate is probably the result of the shorter lengths of the poly-G tracts associated with PV in *Campylobacter* species, which can be accurately sequenced on the Illumina platform. Nevertheless, we have observed that poorly sequenced genomes with large numbers of contigs (>400) can result in reduced or inaccurate detection of SSR PV loci (Fig. 5 and data not shown). In conclusion, coverage of the SSR PV loci in the genus *Campylobacter* is likely to be high as all of the genome sequences were derived on the Illumina platform and the majority either were closed whole genome sequences or contained low numbers of contigs in the assembled sequences (Table S5).

The majority of phase-variable genes identified were found only in a small number of genome sequences or have many non-PV homologues, indicating that PV has evolved multiple times. Previous literature has suggested that the evolution of PV requires on-going selection for both the ON and the OFF states [44, 45]. While this process may be acting on conserved PV genes, it is possible that rare PV genes or alleles arise by chance and that purifying selection has not had sufficient time to remove the SSR. Four possibilities for the initial genesis of phase-variable loci are: equivalent amino acid coding sequences mutate to produce an SSR by neutral drift (i.e. three consecutive glycines become 9Gs); a shorter tract lengthens to become a variable tract; recombination transfers the SSR to a new gene; and finally, random mutation occurs to generate a mutable sequence. In *Campylobacter*, the low G/C content (i.e. a median of 30.4 % for genomes deposited in NCBI) limits the potential for random mutations to produce the poly-G tracts found in most phase-variable genes. Analysis of non-PV homologues in the output of Phasome*It* provides evidence of disrupted tracts (i.e. GGGGGGAGGG) where a point mutation could potentially create a phase-variable tract, or conversely where a point mutation has inactivated an existing phase-variable tract. There are also genes where the sequence is entirely dissimilar and could have arisen by recombination-mediated insertion of an SSR to produce a new variable locus. Recombination between loci should produce similar sequences surrounding the SSR, but the only evidence of this effect was for *cj1421c* and *cj1422c*, which share an ~1000 bp region of homology and appear to undergo frequent recombination, as evidenced by the high similarity of these regions in multiple strains where both of these genes are present. These regions have also been observed to spontaneously recombine under laboratory conditions [46].

We have identified distinct core phasomes for each of the four most sampled *Campylobacter* species. Core phasomes may exist in the other species but cannot be evaluated due to the small sample sets. Core phasomes are potential indicators of the key selective pressures acting on each species. All genes in the core phasomes were poly-G/C tracts (see Table 1), reiterating the major contributions of these elements to the evolution of PV in camplylobacters. Furthermore, all core phasome genes have poly-G/C tract lengths that have been experimentally demonstrated to facilitate efficient PV in *C. jejuni* [21] and the majority (92 %; 46/50) show variability between homology group members indicative of actual PV. Experimental confirmation of PV of these genes in the non-*C. jejuni* species is required in order to facilitate the evaluation of the adaptive potential of these genes. Despite the similarities between *C. jejuni* and *C. coli*, only four homology groups are shared between these species: *cj0170*, *cj1295*, *maf1* and *maf7*. One group, *cj1295*, is present in the core phasome of *C. fetus*. These four homology groups encode known or putative modifiers of the flagellum [36–38, 43]. As the flagellum mediates both motility and aggregation, PV may permit switching between these opposing functions. Alternatively, PV may facilitate avoidance of phage predation as the flagellum is a known receptor for phage attachment [47]. Each of these four core phasomes contains at least one homology group that is present in all isolates and in multiple copies in each isolate. The conservation and amplification of these PV genes must be driven by strong selection. Confusingly, despite their overall prevalence, no R/M systems are present in the core phasomes. PV of R/M systems in *Campylobacter* may therefore be transient and not subject to stringent selection.

Incorrect annotation is a major consideration when interpreting Phasome*It* data. Studies have indicated that between 8 and 37 % of all genome sequence annotation may be incorrect depending on the stringency of criteria applied [5], meaning that manual inspection of Phasome*It* datasets should be performed carefully to prevent carrying forward of any error. The top ten homology groups were re-interogated by blast, which indicated that a high proportion of the PV homology groups are modifiers of surface structures, including the three largest homology groups. This is consistent with previous reports that describe PV as preventing recognition by the immune system or bacteriophages [48]. Several RM systems were identified within the *Campylobacter* PV homology groups, as seen in a range of species [34, 49–51]. These PV genes may prevent the accumulation of resistant phages [52], increase plasmid transfer or other genetic exchange [53], or cause changes in gene expression through differential DNA methylation. The absence of R/M systems from the core phasome but wide prevalence may reflect requirements for functional diversity in methylation sites to combat the rapid evolution of the phage predators of this genus.

PV genes of *C. jejuni* are known to control expression of virulence and transmission phenotypes such as serum resistance, bacterial aggregation and the structures of the autoantigens responsible for *Campylobacter*-associated Guillian–Barré and Miller–Fisher diseases. We therefore examined whether *Campylobacter* phasomes were associated with human disease or transmission through the food chain using approaches that have been applied to other studies of the pan-genome of which PV genes are a component [54]. A confounder of this study was the potential for linkage of the PV genes with other determinants of these traits due to co-inheritance during clonal evolution. Analysis of multiple *C. jejuni* isolates demonstrated that the presence or absence of particular PV homology groups was associated with ST-complex. This indicated that PV genes are co-evolving with other core genes and there is insufficient horizontal transfer across clonal complexes to abolish covariance. Thus, analysis of associations of PV genes with specific traits required a focus on specific STs and clonal complexes to reduce these confounding effects of linkage. This approach led to detection of a weak association between the phasome and host isolation source for the ST-828 complex of *C. coli*. While more detailed evolutionary reconstruction may be required, our analysis of presence/absence of PV homology groups indicates that disease-causing chicken isolates and non-disease-causing cattle isolates cluster separately. This pattern may have arisen because a particular set of PV genes facilitate chicken colonization or survival through the food production chain for ST-828 isolates or could be an artefact due to sampling bias.

The lack of associations in the other STs may reflect an insufficient number of samples for statistical significance, absence of any role of the phasome in host specialization, or dilution of signal strength as a consequence of frequent movement of these strains between hosts. Alternatively, the large numbers of rare PV groups may play a role in host adaptation, because if these groups substitute for one another in bacterial adaptation to a particular host, this would also dilute any association signal. Finally, micro-environmental factors in host adaptation (e.g. phage colonization with different strains, or differences in the microbiome) may strongly influence the phasome and swamp broader host signals.

Here we have characterized the full breadth of SSR-mediated PV in samples from the genus *Campylobacter* using a newly developed program – Phasome*It.* We show that the majority of these bioinformatically identified PV loci contain poly-G repetitive sequences and encode genes involved in modification of surface structures. We provide insights into the possible genesis of PV loci in this genus through comparison of homologous loci, not containing SSRs. We show that conservation of PV genes is prevalent in several species from this genus, forming a ‘core phasome’ which may suggest common mechanisms of environmental and host adaptation. We also show that there are associations of the phasome with ST, and a weak association with host, providing evidence for further evolution of PV loci in response to fluctuating environmental conditions. Finally, development of Phasome*It* provides a tool for studying SSR-mediated phase variation in myriad other bacterial species.

## Data bibliography

1. Aidley J, Wanford JJ, Green LR, Sheppard SK, Bayliss CD. Github. https://github.com/JackAidley/PhasomeIt/.
2. Aidley J, Wanford JJ, Green LR, Sheppard SK, Bayliss CD. Figshare. https://doi.org/10.6084/m9.figshare.7066475.v1.
3. Aidley J, Wanford JJ, Green LR, Sheppard SK, Bayliss CD. Figshare. https://doi.org/10.6084/m9.figshare.7066484.v1.
4. Aidley J, Wanford JJ, Green LR, Sheppard SK, Bayliss CD. Figshare. https://doi.org/10.6084/m9.figshare.7066487.v1.

## Supplementary Data

Supplementary File 1

## References

[1] van der Woude MW, Bäumler AJ (2004). Phase and antigenic variation in bacteria. Clin Microbiol Rev.

[2] Bayliss CD, Palmer ME (2012). Evolution of simple sequence repeat-mediated phase variation in bacterial genomes. Ann N Y Acad Sci.

[3] Cooper KK, Cooper MA, Zuccolo A, Joens LA (2013). Re-sequencing of a virulent strain of *Campylobacter jejuni* NCTC11168 reveals potential virulence factors. Res Microbiol.

[4] Oldfield NJ, Matar S, Bidmos FA, Alamro M, Neal KR (2013). Prevalence and phase variable expression status of two autotransporters, NalP and MspA, in carriage and disease isolates of *Neisseria meningitidis*. PLoS One.

[5] Aidley J (2017). Genomics and Population Dynamics of Phase Variable Genes In campylobacter.

[6] Wanford JJ, Green LR, Aidley J, Bayliss CD (2018). Phasome analysis of pathogenic and commensal *Neisseria* species expands the known repertoire of phase variable genes, and highlights common adaptive strategies. PLoS One.

[7] Moxon R, Bayliss C, Hood D (2006). Bacterial contingency loci: the role of simple sequence DNA repeats in bacterial adaptation. Annu Rev Genet.

[8] Lecuit M, Abachin E, Martin A, Poyart C, Pochart P (2004). Immunoproliferative small intestinal disease associated with *Campylobacter jejuni*. N Engl J Med.

[9] Tam CC, O'Brien SJ, Adak GK, Meakins SM, Frost JA (2003). *Campylobacter coli* - an important foodborne pathogen. J Infect.

[10] Burgos-Portugal JA, Kaakoush NO, Raftery MJ, Mitchell HM (2012). Pathogenic potential of *Campylobacter ureolyticus*. Infect Immun.

[11] Jones K (2001). *Campylobacters* in water, sewage and the environment. Symp Ser Soc Appl Microbiol.

[12] O'Leary J, Corcoran D, Lucey B (2009). Comparison of the EntericBio multiplex PCR system with routine culture for detection of bacterial enteric pathogens. J Clin Microbiol.

[13] O'Donovan D, Corcoran GD, Lucey B, Sleator RD (2014). *Campylobacter ureolyticus*: a portrait of the pathogen. Virulence.

[14] Gebhart CJ, Edmonds P, Ward GE, Kurtz HJ, Brenner DJ (1985). *"Campylobacter hyointestinalis*" sp. nov.: a new species of Campylobacter found in the intestines of pigs and other animals. J Clin Microbiol.

[15] On SL, Bloch B, Holmes B, Hoste B, Vandamme P (1995). *Campylobacter hyointestinalis* subsp. *lawsonii* subsp. nov., isolated from the porcine stomach, and an emended description of *Campylobacter hyointestinalis*. Int J Syst Bacteriol.

[16] Yahara K, Méric G, Taylor AJ, de Vries SP, Murray S (2017). Genome-wide association of functional traits linked with *Campylobacter jejuni* survival from farm to fork. Environ Microbiol.

[17] Sheppard SK, Didelot X, Meric G, Torralbo A, Jolley KA (2013). Genome-wide association study identifies vitamin B5 biosynthesis as a host specificity factor in *Campylobacter*. Proc Natl Acad Sci USA.

[18] Gilbert MJ, Miller WG, Yee E, Zomer AL, van der Graaf-van Bloois L (2016). Comparative genomics of *Campylobacter fetus* from reptiles and mammals reveals divergent evolution in host-associated lineages. Genome Biol Evol.

[19] Lin WH, Kussell E (2012). Evolutionary pressures on simple sequence repeats in prokaryotic coding regions. Nucleic Acids Res.

[20] Wanford JJ, Lango-Scholey L, Nothaft H, Hu Y, Szymanski CM (2018). Random sorting of *Campylobacter jejuni* phase variants due to a narrow bottleneck during colonization of broiler chickens. Microbiology.

[21] Bayliss CD, Bidmos FA, Anjum A, Manchev VT, Richards RL (2012). Phase variable genes of *Campylobacter jejuni* exhibit high mutation rates and specific mutational patterns but mutability is not the major determinant of population structure during host colonization. Nucleic Acids Res.

[22] Cock PJ, Antao T, Chang JT, Chapman BA, Cox CJ (2009). Biopython: freely available Python tools for computational molecular biology and bioinformatics. Bioinformatics.

[23] Merkel A, Gemmell NJ (2008). Detecting microsatellites in genome data: variance in definitions and bioinformatic approaches cause systematic bias. Evol Bioinform Online.

[24] Benson DA, Cavanaugh M, Clark K, Karsch-Mizrachi I, Lipman DJ (2013). GenBank. Nucleic Acids Res.

[25] Brocchieri L, Karlin S (2005). Protein length in eukaryotic and prokaryotic proteomes. Nucleic Acids Res.

[26] Talevich E, Invergo BM, Cock PJ, Chapman BA (2012). Bio.Phylo: a unified toolkit for processing, analyzing and visualizing phylogenetic trees in Biopython. BMC Bioinformatics.

[27] Thomas DK, Lone AG, Selinger LB, Taboada EN, Uwiera RR (2014). Comparative variation within the genome of *Campylobacter jejuni* NCTC 11168 in human and murine hosts. PLoS One.

[28] Revez J, Schott T, Rossi M, Hänninen ML (2012). Complete genome sequence of a variant of *Campylobacter jejuni* NCTC 11168. J Bacteriol.

[29] Skarp CP, Akinrinade O, Nilsson AJ, Ellström P, Myllykangas S (2015). Comparative genomics and genome biology of invasive *Campylobacter jejuni*. Sci Rep.

[30] Sheppard SK, Cheng L, Méric G, de Haan CP, Llarena AK (2014). Cryptic ecology among host generalist *Campylobacter jejuni* in domestic animals. Mol Ecol.

[31] Parkhill J, Wren BW, Mungall K, Ketley JM, Churcher C (2000). The genome sequence of the food-borne pathogen *Campylobacter jejuni* reveals hypervariable sequences. Nature.

[32] Pearson BM, Rokney A, Crossman LC, Miller WG, Wain J (2013). Complete genome sequence of the *Campylobacter coli* clinical isolate 15-537360. Genome Announc.

[33] Henderson IR, Owen P, Nataro JP (1999). Molecular switches–the ON and OFF of bacterial phase variation. Mol Microbiol.

[34] Saunders NJ, Peden JF, Hood DW, Moxon ER (1998). Simple sequence repeats in the *Helicobacter pylori* genome. Mol Microbiol.

[35] de Bolle X, Bayliss CD, Field D, van de Ven T, Saunders NJ (2000). The length of a tetranucleotide repeat tract in *Haemophilus influenzae* determines the phase variation rate of a gene with homology to type III DNA methyltransferases. Mol Microbiol.

[36] Hitchen P, Brzostek J, Panico M, Butler JA, Morris HR (2010). Modification of the *Campylobacter jejuni* flagellin glycan by the product of the Cj1295 homopolymeric-tract-containing gene. Microbiology.

[37] Karlyshev AV, Linton D, Gregson NA, Wren BW (2002). A novel paralogous gene family involved in phase-variable flagella-mediated motility in *Campylobacter jejuni*. Microbiology.

[38] van Alphen LB, Wuhrer M, Bleumink-Pluym NM, Hensbergen PJ, Deelder AM (2008). A functional *Campylobacter jejuni* maf4 gene results in novel glycoforms on flagellin and altered autoagglutination behaviour. Microbiology.

[39] Sheppard SK, Colles FM, Carthy N, Strachan NJC, Ogden ID (2011). Niche segregation and genetic structure of *Campylobacter jejuni* populations from wild and agricultural host species.

[40] Sheppard SK, Colles F, Richardson J, Cody AJ, Elson R (2010). Host association of *Campylobacter* genotypes transcends geographic variation.

[41] Lango-Scholey L, Aidley J, Woodacre A, Jones MA, Bayliss CD (2016). High throughput method for analysis of repeat number for 28 phase variable loci of *Campylobacter jejuni* strain NCTC11168. PLoS One.

[42] Aidley J, Rajopadhye S, Akinyemi NM, Lango-Scholey L, Jones MA (2017). Nonselective bottlenecks control the divergence and diversification of phase-variable bacterial populations. MBio.

[43] Artymovich K, Kim JS, Linz JE, Hall DF, Kelley LE (2013). A "successful allele" at *Campylobacter jejuni* contingency locus Cj0170 regulates motility; "successful alleles" at locus Cj0045 are strongly associated with mouse colonization. Food Microbiol.

[44] Libby E, Rainey PB (2011). Exclusion rules, bottlenecks and the evolution of stochastic phenotype switching. Proc Biol Sci.

[45] Patra P, Klumpp S (2015). Emergence of phenotype switching through continuous and discontinuous evolutionary transitions. Phys Biol.

[46] McNally DJ, Lamoureux MP, Karlyshev AV, Fiori LM, Li J (2007). Commonality and biosynthesis of the O-methyl phosphoramidate capsule modification in *Campylobacter jejuni*. J Biol Chem.

[47] Sørensen MC, van Alphen LB, Harboe A, Li J, Christensen BB (2011). Bacteriophage F336 recognizes the capsular phosphoramidate modification of *Campylobacter jejuni* NCTC11168. J Bacteriol.

[48] Aidley J, Sørensen MCH, Bayliss CD, Brøndsted L (2017). Phage exposure causes dynamic shifts in the expression states of specific phase-variable genes of *Campylobacter jejuni*. Microbiology.

[49] Ryan KA, Lo RY (1999). Characterization of a CACAG pentanucleotide repeat in *Pasteurella haemolytica* and its possible role in modulation of a novel type III restriction-modification system. Nucleic Acids Res.

[50] Anjum A, Brathwaite KJ, Aidley J, Connerton PL, Cummings NJ (2016). Phase variation of a Type IIG restriction-modification enzyme alters site-specific methylation patterns and gene expression in *Campylobacter jejuni* strain NCTC11168. Nucleic Acids Res.

[51] Manso AS, Chai MH, Atack JM, Furi L, de Ste Croix M (2014). A random six-phase switch regulates pneumococcal virulence via global epigenetic changes. Nat Commun.

[52] Bayliss CD, Callaghan MJ, Moxon ER (2006). High allelic diversity in the methyltransferase gene of a phase variable type III restriction-modification system has implications for the fitness of *Haemophilus influenzae*. Nucleic Acids Res.

[53] Seib KL, Peak IR, Jennings MP (2002). Phase variable restriction-modification systems in *Moraxella catarrhalis*. FEMS Immunol Med Microbiol.

[54] Snipen L, Ussery DW (2010). Standard operating procedure for computing pangenome trees. Stand Genomic Sci.

