# Supplementary material for: PhasomeIt: an ‘omics’ approach to cataloguing the potential breadth of phase variation in the genus Campylobacter
Source: Microb Genom. 2018 Oct 23;4(11):e000228. doi: 10.1099/mgen.0.000228 (PMC6321876; doi:10.1099/mgen.0.000228)
Supplement: Supplementary File 1 [file mgen-4-228-s001.pdf]

Supplementary Table 1. Speed comparison for Bossref

Program[1]	Time (s)[2]
Bossref	5.9
TROLL	19.9

[1]Bossref, this manuscript; TROLL (Castelo *et al.*, 2002). [2]Timings based on search for all perfect SSRs of 20bp or greater and repeat unit size of 1-5bp in the *Arabidopsis thaliana* genome sequence. Performed on BioLinux 8.0.7 running in Oracle VirtualBox 5.1.2 with 4 Gb of RAM on an Intel i5-6600K processor running at 4.2 Ghz. Timings are the average of 5 runs of each program.

Supplementary Table 2. Accession numbers for one or more genome sequences of 15 *Campylobacter* species.

Species	n	Strains (accession numbers)
C. coli	10	RM1875 (CP007183), RM4661 (CP007181), RM5611 (CP007179), FB1 (CP011015), BFR-CA-9557 (CP011777), HC2-48 (CP013034), OR12 (CP013733), CVM N29710 (CP004066), YH501 (CP015528), 15-537360 (CP006702)
C. concisus	2	13826 (CP000792), ATCC 33237 (CP012541)
C. curvus	1	525.92 (CP000767)
C. fetus	8	04/554 (CP008808), 97/608 (CP008810), pet-3 (CP009226), 84-112 (HG004426), 82-40 (CP000487), SP3 (CP010953), 03-427 (CP006833), cfvi03/293 (CP006999)
C. gracilis	1	ATCC 33236 (CP012196)
C. hominis	1	ATCC BAA-381 (CP000776)
C. hyointestinalis	2	LMG 9260 (CP015575), CCUG 27631 (CP015576)
C. iguaniorum	3	1485E (CP009043), 2463D (CP010995), RM11343 (CP015577)
C. insulaenigrae	1	NCTC 12927 (CP007770)

C. jejuni	35	R14 (CP005081), CG8421 (CP005388), MTVDSCj20 (CP008787), 00-2538 (CP006707), 00-2425 (CP006729), 00-2544 (CP006709), 00-2426 (CP006708), F38011 (CP006851), YH001 (CP010058), 00-1597 (CP010306), 00-6200 (CP010307), 01-1512 (CP010072), 00-0949 (CP010301), ICDCCJ07001 (CP002029), 35925B2 (CP010906), NCTC 11168 (AL111168), RM1221 (CP000025), 81-176 (CP000538), M1 (CP001900), S3 (CP001960), Isolate: IA3902 (CP001876), 269.97 (CP000768), 81116; NCTC 11828 (CP000814), 32488 (CP006006), RM3197 (CP012689), RM3196 (CP012690), CJM1cam (CP012149), NCTC11351 (LN831025), CJ677CC519 (CP010471), RM3194 (CP014344), OD267 (CP014744), WP2202 (CP014742), PT14 (CP003871), RM1285 (CP015209), 4031 (HG428754)
C. lari	7	LMG 11760 (CP007771), NCTC 11845 (CP007775), RM16701 (CP007777), RM16712 (CP007778), Slaughter Beach (CP011372), RM2100; ATCC BAA-1060D (CP000932), CCUG 22395 (CP007776)
C. peloridis	1	LMG 23910 (CP007766)
C. sp.	1	RM16704 (CP007769)
C. subantarcticus	2	LMG 24374 (CP007772), LMG 24377 (CP007773)
C. ureolyticus	1	RIGS 9880 (CP012195)

Supplementary Table 3. The twenty most frequent phase-variable functional groups.

Group name	In frame[1]	Total[2] PV	Total[2]	Putative function
maf7	200	203	233	carbonic anyhydrase
cj1295	64	65	72	hypothetical protein (DUF2172 domain), putative M28 family zinc peptidase
maf1	63	65	123	motility accessory factor
cj0170	47	52	54	SAM-dependent methyltransferase
cj1421c	45	52	55	putative sugar transferase
ubiE_3	48	48	48	SAM-dependent methyltransferase
cj0045c	43	43	45	Hemerythrin-like iron-binding protein
cipA	36	36	39	Invasion protein CipA
A911_07000	34	34	50	sugar transferase
UPTC4110_0710	30	31	45	No annotation data
cj1296	29	30	92	aminoglycoside N3'-acetyltransferase
hxuB_1	0	25	36	Heme/hemopexin transporter protein HuxB precursor
ansA	0	24	78	L-asparaginase
cj0628	19	23	35	putative lipoprotein
cjeI	21	21	47	restriction endonuclease
lgrA	21	21	29	formyl transferase domain protein

A911_t08342	0	21	75	No annotation data
CFT03427_1115	19	20	21	autotransporter domain protein
PJ18_06805	18	20	50	N-acetyl sugar amidotransferase
epsM	0	19	23	putative transferase

[1]In frame, the number of genes with an SSR in the CDS; [2]Total PV, total number of genes with either an SSR in the CDS or in an intergenic region.

.

Supplementary Table 4. Number coding of complete *Campylobacter* genome sequences.

Number	Species	Strain
1		NCTC 11168
2		81-176
3		PT14
4		00-0949
5		00-1597
6		00-2425
7		00-2426
8		00-2538
9	*C. jejuni*	00-2544
10		00-6200
11		01-1512
12		269.97
13		4031
14		32488
15		35925B2
16		81116; NCTC 11828
17		CG8421

18		CJ677CC519
19		CJM1cam
20		F38011
21		IA3902
22		ICDCCJ07001
23		M1
24		MTVDSCj20
25		NCTC11351
26		OD267
27		R14
28		RM1221
29		RM1285
30		RM3194
31		RM3196
32		RM3197
33		S3
34		WP2202
35		YH001
36	*C. coli*	15-537360

37		BFR-CA-9557
38		CVM N29710
39		FB1
40		HC2-48
41		OR12
42		RM1875
43		RM4661
44		RM5611
45		YH501
46	*C. concisus*	13826
47		ATCC 33237
48	*C. curvus*	525.92
49		03-427
50		04/554
51		82-40
52	*C. fetus*	84-112
53		97/608
54		SP3
55		cfvi03/293

56		pet-3
57	*C. gracilis*	ATCC 33236
58	*C. hominis*	ATCC BAA-381
59	*C. hyointestinalis*	CCUG 27631
60		LMG 9260
61	*C. iguaniorum*	1485E
62		2463D
63		RM11343
64	*C. insulaenigrae*	NCTC 12927
65	*C. lari*	CCUG 22395
66		LMG 11760
67		NCTC 11845
68		RM2100; ATCC BAA-1060D
69		RM16701
70		RM16712
71		Slaughter Beach
72	*C. peloridis*	LMG 23910
73	*C. sp.*	RM16704

74	*C. subantarcticus*	LMG 24374
75		LMG 24377
76	*C. ureolyticus*	RIGS 9880
77	*C. volucris*	LMG 24379

Supplementary Table 5. Numbers of contigs, PV loci and homology groups for the partial genome sequences of *C. jejuni* and *C. coli* isolates with meta-data

Species	Genome Sequence File No.	No. of Contigs	No. of PV Genes	No. of PV Homology Groups
C. jejuni	NCTC 11168	1	31	25
C. coli	15-537360	1	21	17
C. coli - clade 2	1.gbk	167	10	9
C. coli - clade 1	2.gbk	184	17	13
C. coli - clade 3	3.gbk	125	4	4
C. jejuni	4.gbk	108	7	7
C. coli - clade 1	5.gbk	255	10	6
C. coli - clade 3	8.gbk	281	3	3
C. coli - clade 2	10.gbk	191	13	11
C. coli - clade 2	11.gbk	312	9	9
C. coli - clade 2	12.gbk	282	8	8
C. jejuni	13.gbk	86	13	13
C. jejuni	14.gbk	324	12	12
C. coli - clade 1	15.gbk	216	11	11
C. coli - clade 1	17.gbk	79	11	8
C. coli - clade 1	18.gbk	206	16	14
C. coli - clade 1	19.gbk	276	10	10
C. coli - clade 1	20.gbk	691	10	9
C. coli - clade 1	21.gbk	374	6	5
C. jejuni	22.gbk	169	7	7
C. jejuni	23.gbk	446	1	1
C. coli - clade 1	24.gbk	532	10	7
C. coli - clade 1	25.gbk	261	16	15
C. jejuni	26.gbk	1	18	17
C. jejuni	27.gbk	1	20	17
C. jejuni	28.gbk	1	27	25
C. jejuni	29.gbk	1	31	25
C. jejuni	30.gbk	465	14	12

C. jejuni	32.gbk	92	19	17
C. jejuni	34.gbk	91	20	15
C. jejuni	36.gbk	47	27	18
C. jejuni	37.gbk	113	27	24
C. jejuni	39.gbk	84	20	15
C. jejuni	40.gbk	195	21	14
C. jejuni	42.gbk	41	17	14
C. jejuni	43.gbk	3226	31	20
C. coli	44.gbk	55	19	15
C. jejuni	45.gbk	72	13	12
C. jejuni	48.gbk	62	29	25
C. jejuni	49.gbk	181	31	26
C. jejuni	52.gbk	102	18	18
C. jejuni	54.gbk	241	21	15
C. jejuni	55.gbk	271	14	14
C. jejuni	56.gbk	62	20	17
C. jejuni	57.gbk	158	11	10
C. jejuni	59.gbk	60	28	22
C. jejuni	60.gbk	58	29	22
C. jejuni	62.gbk	100	27	24
C. jejuni	63.gbk	139	29	24
C. jejuni	64.gbk	113	14	13
C. jejuni	65.gbk	112	34	29
C. jejuni	66.gbk	129	31	29
C. jejuni	67.gbk	143	20	17
C. jejuni	68.gbk	251	33	31
C. jejuni	69.gbk	144	21	16
C. jejuni	70.gbk	343	22	21
C. jejuni	71.gbk	84	24	18
C. jejuni	72.gbk	63	24	18
C. jejuni	73.gbk	102	27	21
C. jejuni	74.gbk	65	22	19

C. jejuni	75.gbk	76	26	20
C. jejuni	77.gbk	65	24	18
C. jejuni	78.gbk	67	23	19
C. jejuni	79.gbk	120	15	12
C. jejuni	80.gbk	73	21	20
C. jejuni	81.gbk	106	14	14
C. jejuni	82.gbk	163	16	15
C. jejuni	83.gbk	617	19	18
C. jejuni	84.gbk	58	16	15
C. jejuni	85.gbk	93	22	18
C. jejuni	86.gbk	197	20	20
C. jejuni	87.gbk	62	30	26
C. jejuni	88.gbk	72	29	23
C. jejuni	89.gbk	130	24	19
C. jejuni	90.gbk	64	15	15
C. jejuni	91.gbk	47	17	17
C. jejuni	92.gbk	98	20	19
C. jejuni	94.gbk	82	31	28
C. jejuni	96.gbk	922	36	27
C. jejuni	97.gbk	114	22	18
C. coli - clade 1	98.gbk	119	23	17
C. jejuni	99.gbk	64	30	24
C. jejuni	100.gbk	192	13	12
C. jejuni	102.gbk	63	16	16
C. jejuni	103.gbk	70	17	17
C. jejuni	104.gbk	115	15	14
C. jejuni	105.gbk	75	28	28
C. jejuni	106.gbk	58	27	25
C. jejuni	107.gbk	273	35	31
C. jejuni	108.gbk	80	32	27
C. jejuni	109.gbk	152	20	16
C. jejuni	110.gbk	72	26	21

C. jejuni	111.gbk	80	16	15
C. jejuni	112.gbk	55	17	16
C. jejuni	113.gbk	72	25	23
C. jejuni	114.gbk	79	14	14
C. jejuni	116.gbk	65	28	22
C. jejuni	117.gbk	113	25	20
C. jejuni	119.gbk	66	16	16
C. jejuni	122.gbk	52	10	10
C. jejuni	124.gbk	112	12	12
C. jejuni	125.gbk	61	14	12
C. jejuni	126.gbk	149	24	22
C. jejuni	127.gbk	2598	4	2
C. jejuni	128.gbk	53	11	11
C. jejuni	129.gbk	143	28	24
C. jejuni	130.gbk	108	31	25
C. jejuni	131.gbk	60	19	16
C. coli - clade 1	132.gbk	122	18	15
C. coli	133.gbk	92	14	12
C. coli - clade 1	134.gbk	77	10	9
C. coli	135.gbk	198	17	14
C. coli - clade 1	136.gbk	150	16	13
C. coli - clade 1	137.gbk	148	12	12
C. coli - clade 1	138.gbk	104	17	14
C. coli - clade 1	139.gbk	243	17	13
C. coli - clade 1	140.gbk	217	18	15
C. coli - clade 1	141.gbk	199	15	13
C. coli - clade 1	142.gbk	118	19	16
C. coli - clade 1	143.gbk	238	15	12
C. coli - clade 1	144.gbk	99	11	9
C. coli - clade 1	145.gbk	125	17	15
C. coli - clade 1	146.gbk	129	17	17
C. coli - clade 1	147.gbk	112	17	14

C. coli - clade 1	148.gbk	116	22	18
C. coli - clade 1	149.gbk	116	22	18
C. coli - clade 1	150.gbk	111	19	17
C. coli - clade 1	151.gbk	110	20	18
C. coli - clade 1	152.gbk	115	15	13
C. coli - clade 1	153.gbk	181	13	12
C. coli - clade 1	154.gbk	146	19	16
C. coli - clade 1	155.gbk	166	16	14
C. coli - clade 1	156.gbk	76	10	10
C. coli - clade 1	157.gbk	161	15	13
C. coli - clade 1	158.gbk	103	17	16
C. coli - clade 1	159.gbk	105	16	14
C. coli - clade 1	160.gbk	157	17	15
C. coli - clade 1	161.gbk	85	15	13
C. coli - clade 1	162.gbk	83	17	14
C. coli - clade 1	163.gbk	169	13	13
C. coli	164.gbk	88	13	11
C. coli - clade 1	165.gbk	80	18	16
C. coli	166.gbk	159	21	16
C. coli - clade 1	167.gbk	127	11	10
C. coli - clade 1	168.gbk	139	14	13
C. coli - clade 1	169.gbk	71	14	12
C. coli - clade 1	170.gbk	110	17	13
C. coli - clade 1	171.gbk	82	13	11
C. jejuni	172.gbk	98	14	13
C. jejuni	173.gbk	195	24	21
C. jejuni	174.gbk	162	14	13
C. jejuni	175.gbk	84	15	13
C. jejuni	176.gbk	101	21	20
C. jejuni	177.gbk	127	16	15
C. jejuni	178.gbk	108	19	19
C. jejuni	179.gbk	116	23	19

C. jejuni	180.gbk	151	27	24
C. jejuni	181.gbk	89	13	13
C. jejuni	182.gbk	109	17	17
C. jejuni	183.gbk	114	18	17
C. jejuni	184.gbk	116	12	12
C. jejuni	185.gbk	221	23	21
C. jejuni	186.gbk	88	19	17
C. jejuni	187.gbk	145	21	19
C. jejuni	188.gbk	88	20	20
C. jejuni	189.gbk	129	15	14
C. jejuni	190.gbk	92	20	18
C. jejuni	191.gbk	102	13	13
C. jejuni	192.gbk	170	26	23
C. jejuni	193.gbk	86	22	21
C. jejuni	194.gbk	214	23	21
C. jejuni	195.gbk	107	21	17
C. jejuni	196.gbk	117	21	19
C. jejuni	197.gbk	84	19	17
C. jejuni	198.gbk	214	24	24
C. jejuni	199.gbk	90	17	16
C. jejuni	200.gbk	183	25	22
C. jejuni	201.gbk	189	27	26
C. jejuni	202.gbk	102	16	13
C. jejuni	203.gbk	94	15	12
C. jejuni	204.gbk	179	25	23
C. jejuni	205.gbk	93	20	18
C. jejuni	206.gbk	111	19	19
C. jejuni	207.gbk	138	24	22
C. jejuni	208.gbk	94	17	15
C. jejuni	209.gbk	80	18	16
C. jejuni	210.gbk	89	26	24
C. jejuni	211.gbk	174	18	17

C. jejuni	212.gbk	76	17	15
C. jejuni	213.gbk	117	24	22
C. jejuni	214.gbk	85	21	19

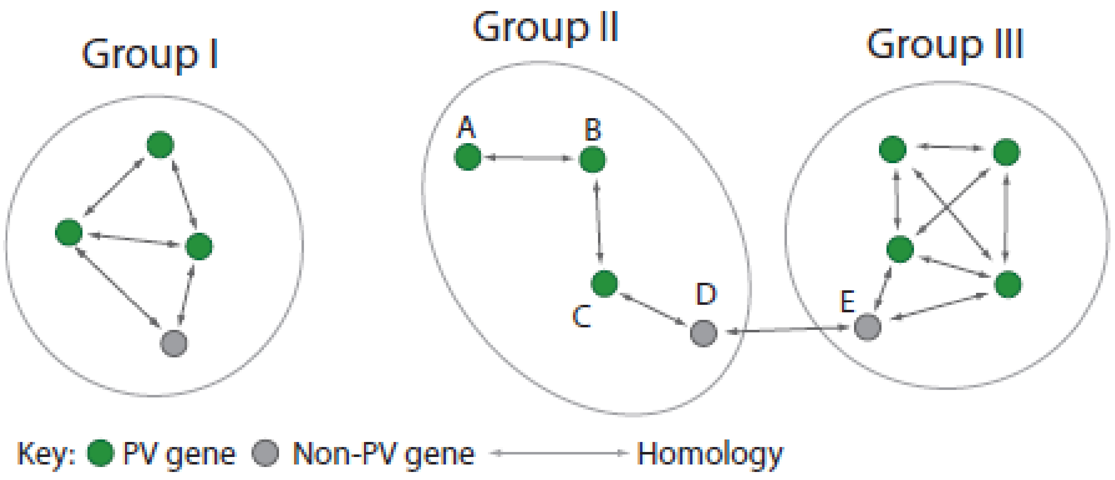

Supplementary Figure 1. Grouping genes into homology groups. Coloured circles show identified genes, and arrows show homology relationships between genes. Genes that are connected by homology relationships are placed in the same group. Thus, A and C are in the same homology group even though they share no homology relationship because they are both homologous to B. D and E do not connect groups II and III because D and E are non-PV homologues and the program does not search for homology relationships from these genes. Instead they are grouped only with PV genes homologous to them.

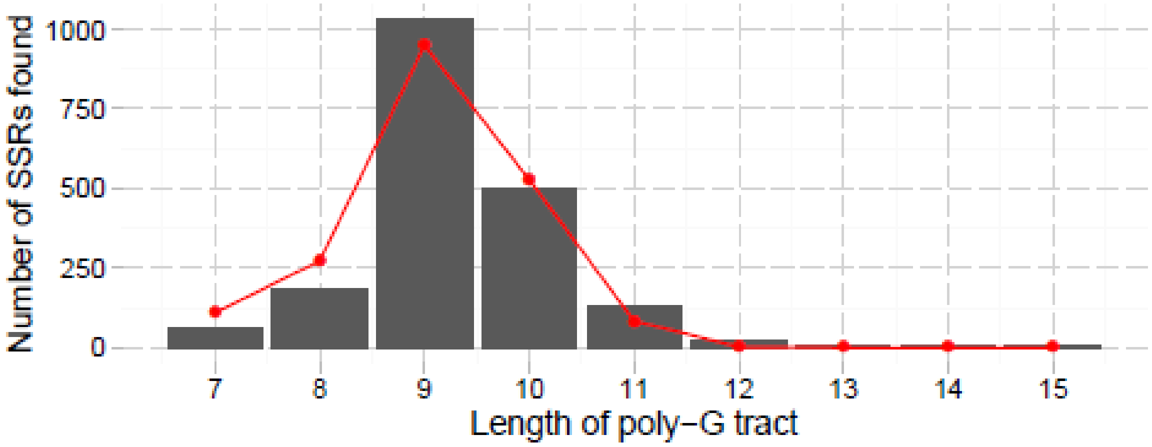

Supplementary Figure 2. Distribution of poly-G tract lengths in *Campylobacter* genome sequences. This graph shows counts of poly-G tracts of each length found in the analysed *Campylobacter* genome sequences from a total of 1,944 tracts. Red line shows predicted distribution without selection based on empirical data of mutation rate for each tract length. Poly-G tracts of length below 7 were excluded from the search. Tracts that are poly-C in the direction of coding are also included.

Shorter tract (*clpX* group):

```
              I  I  E  G  S  L  V  N  I  P  P  R  G  G  R  K  H  P  N  Q  E  F
NCTC 11168   ATTATCGAAGGAAGTTTGGTAAATATTCCACCAAGGGGGGGGAGAAAACATCCAAATCAAGAGTTT
             ||||||||||||||||||||||||||||||||||..|||||.||||||||||||||||||||||||
81-176       ATTATCGAAGGAAGTTTGGTAAATATTCCACCAAAAGGGGGAAGAAAACATCCAAATCAAGAGTTT
              I  I  E  G  S  L  V  N  I  P  P  K  G  G  R  K  H  P  N  Q  E  F
```

Disrupted tract (*CJJ81176_0758* group):

```
              L  I  T  A  A  F  T  D  R  G  G  G  F  N  N  Q  T  I  L  K  E  N
4031         TTAATTACTGCGGCGTTTACTGACAGGGGGGGGGGATTTAACAATCAAACCATTTTAAAAGAAAAC
             |||||||||||||||||||||||||||||||.||||||||||||||||||||||||||||||||||
NCTC 11168   TTAATTACTGCGGCGTTTACTGACAGGGGGGAGGGATTTAACAATCAAACCATTTTAAAAGAAAAC
              L  I  T  A  A  F  T  D  R  G  E  G  F  N  N  Q  T  I  L  K  E  N
```

Dissimilar sequence (*kfoC* group):

```
              G  L  L  R  A  R  Y  E  G  V  K  A  A  G  G  G  Y  I  M  F  L  D
NCTC 11351   GGTCTTTTAAGAGCTAGATATGAAGGAGTTAAGGCAGCTGGGGGGGGATATATTATGTTTTTAGAC
             ||||||||||||||.|||||||||||.||.||.|.|||.        .|||||.|||||||||||.
81-176       GGTCTTTTAAGAGCAAGATATGAAGGTGTGAAAGTAGCAAACTCTCCTTATATAATGTTTTTAGAT
              G  L  L  R  A  R  Y  E  G  V  K  V  A  N  S  P  Y  I  M  F  L  D
```

Supplementary Figure 3. Comparison of region around PV tract against non-PV homologues. This figure shows examples of three possible cases of how PV and non-PV homologues differ. In each case, the PV homologue is shown on the top with the translated sequence above, and the non-PV homologue is shown below with the translated sequence below. Amino acid sequence differences are highlighted in red. All three are drawn from strains of *C. jejuni*.
